# Accelerating regulation in response to COVID-19

**DOI:** 10.2471/BLT.20.020820

**Published:** 2020-08-01

## Abstract

Regulators are finding ways to support the expedited development and distribution of novel coronavirus-related vaccines, tests and treatments. Gary Humphreys reports.

Dr Andrea Julsing Keyter has never been so busy. A senior manager at the South African Health Products Regulatory Authority (SAHPRA), in Pretoria, South Africa, Keyter has seen a sharp increase in her workload since April, mostly due to medical products designed to prevent exposure to, test for or treat novel coronavirus disease (COVID-19).

“We have received approximately 250 [medical product] applications per month since April 2020 compared to an average of approximately 20 per month,” she says. “SAHPRA has also been collaborating with the national reference laboratory to conduct performance evaluations on serological antibody test kits and molecular test kits, and participating in webinars aimed at supporting the local manufacture of personal protective equipment, sanitisers, diagnostics and ventilators.”

On top of everything else, SAHPRA is actively investigating unauthorised importation of test kits, masks and thermometers, and has conducted numerous site visits.

“It would be fair to say the COVID-19 pandemic is keeping us all pretty occupied,” Keyter says.

Keyter is one of a host of regulators faced with the challenge of not only assessing, and – where appropriate – approving new products, but doing it quickly enough to make a difference to the COVID-19 pandemic response in the coming months.

This is a major challenge, especially for national regulatory authorities (NRAs) in low- and middle-income countries, which often lack the resources they need to fulfil their missions.

“It has been estimated that around a third of NRAs lack the capacity to perform even core regulatory functions such as product assessment,” says Dr Samvel Azatyan, a regulation and safety expert at the World Health Organization (WHO).

Focusing solely on medicines and vaccines, it has been estimated that the total time to registration in low- and middle-income countries (including product approval in the country of manufacture, WHO prequalification and approval from the regulatory agency in the country for which marketing permission has been requested) is on average 4–7 years after completion of phase-3 trials and assembly of a dossier for marketing application.

“In public health emergencies, such as the one we are currently going through, regulators are expected to act fast,” says Azatyan, pointing out that regulators have been called on to support the Access to COVID-19 Tools (ACT) Accelerator initiative, a global collaboration that was launched in April and is designed to accelerate the development, production and equitable distribution of new COVID-19 essential health technologies as they become available.

“Lessons from previous public health emergencies have triggered early action.”

Socorro Escalante

Regulators are striving to meet the acceleration challenge in a variety of ways, but a common thread through all of them is the introduction of increased regulatory flexibility.

Regulatory flexibility is not a new concept and underpins a variety of accelerated approval and adaptive licensing mechanisms, some of which have been a part of the regulatory landscape for more than 20 years. For example, the United States of America’s (USA) Food and Drug Administration’s (FDA) Fast Track process – which, as the name suggests, is a regulatory mechanism designed to expedite the review of certain drugs – was launched in 1997.

However, pressure to develop new medical products as part of the COVID-19 response, is pushing regulators to go further.

Just how far they should be ready to go was part of a discussion that took place at an on-line meeting of the International Coalition of Medicines Regulatory Authorities (ICMRA) in March.

Co-chaired by the European Medicines Agency (EMA) and the FDA, the meeting was attended by delegates from 17 countries, representing more than 20 medicines regulatory authorities who were joined by experts from WHO and the European Commission to discuss regulatory considerations related to the development of novel coronavirus (SARS-CoV-2) vaccine candidates.

One of the topics discussed at the meeting was whether human trials of vaccines could begin without first completing animal studies.

Animal studies perform a vital risk assessment function, allowing researchers to determine whether products are safe and effective. For obvious reasons they usually precede human trials. Animal studies are of vital importance, but they can also add years to vaccine development timelines.

According to an ICMRA report of the 18 March meeting, while not unanimous, participants generally agreed that some vaccine constructs for which there is adequate knowledge around the technology used and the immune response elicited, may be allowed to proceed to human trials.

However, participants also agreed that where human trials are allowed without prior animal studies, such studies are, in general, expected to be conducted in parallel with human trials, so that the data generated are available prior to enrolling large numbers of human subjects into Phase 2 and 3 clinical trials.

The biotechnology company Moderna, working in collaboration with the National Institute of Allergy and Infectious Diseases (NIAID) in the USA has already taken advantage of this flexibility, running animal trials in parallel with Phase 1 human trials of its messenger-RNA vaccine, mRNA 1273.

For their part, NRAs are looking to expedite regulatory processes through information- and work-sharing arrangements that in some cases extend to regulatory reliance, whereby an NRA will rely on the work of better resourced or more established NRAs sometimes referred to as stringent regulatory authorities (SRAs).

WHO has long promoted and supported such collaborative approaches, which not only enhance regulatory effectiveness, but also, by avoiding duplication of effort, speed things up. For example, WHO helped set up a Regional Alliance of National Regulatory Authorities in the WHO Western Pacific Region and WHO Member States approved a regional framework for regulatory strengthening, cooperation and convergence in 2018.

“We consider reliance a useful option.”

Andrea Julsing Keyter

“Regulations play a key part in ensuring that medical products can be made available to the people who need them in a timely fashion and regulatory reliance can help with that,” says Dr Socorro Escalante, a health technologies expert at the WHO Regional Office for the Western Pacific, adding that at least eight Western Pacific Region countries have passed legislation permitting reliance on SRAs within and outside the region.

In Africa too, countries are working together to increase regulatory efficacy and to expedite the review process. These include the 23 members of the African Vaccine Regulatory Forum (AVAREF) a continental platform for regulation of clinical trials that promotes joint reviews and work sharing.

AVAREF recently updated its joint review guidelines recommending a timeline of 10 working days for the processing of COVID-19-related clinical trial assessment, where the product is already registered and being repurposed, and 15 working days for novel products.

According to SAHPRA’s Keyter, South Africa (an AVAREF member), frequently relies on approvals from SRAs, including the FDA and EMA, but also Singapore and Korea, among others.

“We consider reliance a useful option,” she says. “It allows us to retain regulatory sovereignty, avoids duplication of effort and frees up resources for other regulatory functions, including pharmacovigilance, which will be particularly important with the novel COVID-19 medicines and vaccines that may emerge in the coming year.”

Keyter also relies on WHO’s Emergency Use Listing (EUL), which was launched in 2014 in response to the West Africa Ebola virus epidemic and provides a time-limited listing for unlicensed products during Public Health Emergencies of International Concern and in other public health emergencies, where appropriate.

WHO used the EUL for diagnostic kits during the 2014 Ebola virus disease and 2016 Zika virus outbreaks, and applications for listing have been opened to candidate diagnostic kits to detect SARS-CoV-2.

The main purpose of the EUL is to make safe and effective unlicensed medical products available quickly to support United Nations procurement agencies and Member States, but the listing is also of considerable interest to makers of those products.

“Technology innovators see the EUL as an important preliminary validation of safe and effective technologies,” says Jamie Bay Nishi, director of the Global Health Technologies Coalition.

“We look forward to seeing the procedure applied to therapeutics and vaccines as new COVID-19 products are developed or established products are adapted to tackle the pandemic,” she adds.

Emergency use listing is also an option for national regulatory authorities, many of which are introducing their own emergency listing procedures as part of facilitated entry of medical products in public health emergencies.

For Escalante such approaches will be key to ensuring expedited access to COVID-19 tests, treatments and vaccines should these emerge.

“Lessons from previous public health emergencies have triggered early action in the region,” she says, pointing out that the regional office started working on regulatory preparedness in support of the COVID-19 response back in January. It is to be hoped that by January of next year, regulators will have some powerful new products to assess.

**Figure:**
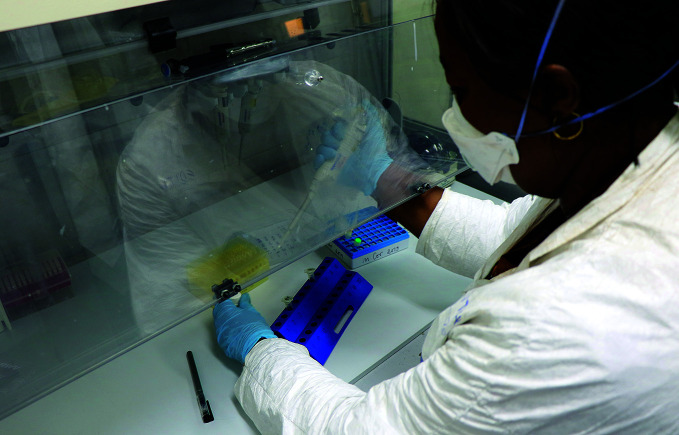
A microbiologist at the National Institute of Biomedical Research in the Democratic Republic of the Congo tests for COVID-19.

WHO/Eugene Kabambi

**Figure:**
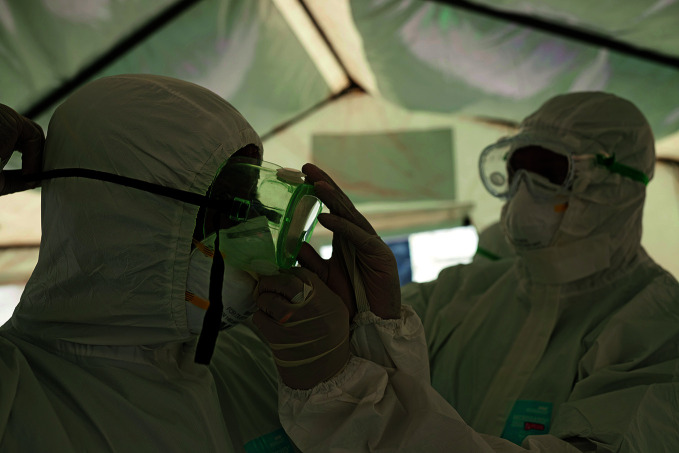
Health workers check personal protective equipment at the isolation centre in Bole Chefe in Addis Ababa, Ethiopia.

WHO/Otto Bakano

